# Chrononutrition and Prenatal Mental Health: The Relationship Between Food Intake Indicators and Prenatal Depressive Symptomatology

**DOI:** 10.3390/nu18213648

**Published:** 2026-11-02

**Authors:** Christina M. Personette, David Phan, Daisy Duan, Namhyun Kim, Kaleab Z. Abebe, Christina M. Scifres, Tina Costacou, Patrick M. Catalano, Hyagriv Simhan, Esa M. Davis, Dara D. Mendez, Marquis S. Hawkins

**Affiliations:** 1 Department of Psychology, University of Pittsburgh, Pittsburgh, PA 15213, USAmah400@pitt.edu (M.S.H.); 2 Division of Endocrinology, Diabetes and Metabolism, Department of Medicine, School of Medicine, Johns Hopkins University, Baltimore, MD 21205, USA; 3 Department of Epidemiology, University of Pittsburgh, Pittsburgh, PA 15261, USA; 4 Department of Medicine, University of Pittsburgh, Pittsburgh, PA 15213, USA; 5 Department of Obstetrics and Gynecology, Indiana University School of Medicine, Indianapolis, IN 46202, USA; 6 Reproductive Endocrine Unit, Department of Medicine, Massachusetts General Hospital, Harvard Medical School, Boston, MA 02114, USA; 7 Department of Obstetrics, Gynecology & Reproductive Sciences, University of Pittsburgh, Pittsburgh, PA 15213, USA; 8 Department of Family and Community Medicine, University of Maryland School of Medicine, Baltimore, MD 21201, USA

**Keywords:** chrononutrition, diet, prenatal depression, perinatal mental health, pregnancy, food timing

## Abstract

Objective: We examined cross-sectional associations between mid-pregnancy food intake indicators and prenatal depressive symptomatology. Methods: This secondary analysis of the Comparison of Two Screening Strategies for Gestational Diabetes (GDM2) trial (*N* = 716) examined five domains of mid-pregnancy food intake (direct timing, energy timing, meal/snack structure, meal energy distribution, and diet quality) derived from 24 h dietary recalls. Depressive symptoms were measured with the Edinburgh Postnatal Depression Scale (EPDS). Generalized linear models examined associations between food intake indicators and both total and high (EPDS ≥ 13) depressive symptoms. Results: The mean (SD) EPDS score was 6.3 (4.9); a total of 12.4% (*n* = 89) of subjects had high depressive symptoms. Eating frequency (exp(B) = 1.084 [1.021, 1.151], *p* = 0.008), snack frequency (exp(B) = 1.063 [1.004, 1.126], *p* = 0.037), nighttime snacking frequency (exp(B) = 1.060 [1.003, 0.121], *p* = 0.040), and total daily energy intake (exp(B) = 1.058 [1.002, 1.118], *p* = 0.044) were positively associated with total depressive symptoms. Energy intake from breakfast (PR = 1.178 [1.029, 1.349], *p* = 0.017) was associated with higher prevalence of high depressive symptoms. Energy intake from dinner (PR = 0.809 [0.695, 0.943], *p* = 0.007), a later timing of the first eating episode (PR = 0.830 [0.699, 0.986], *p* = 0.034) and the first energy quartile (PR = 0.837 [0.702, 0.998], *p* = 0.048) were associated with lower prevalence of high depressive symptoms. Conclusions: These findings extend the prior chrononutrition–depression literature to the prenatal period, implicating eating frequency, energy intake, and meal energy timing and distribution in depressive symptomatology during pregnancy, and warrant further longitudinal investigation.

## 1. Introduction

Rates of depression and anxiety symptoms reach up to 20% in US pregnant populations [1,2]. These high rates are concerning, as they contribute to poor household functioning, relationship difficulties, lower quality of life, suicidal ideation, and impaired child attachment post-delivery [3]. Several studies indicate that the negative outcomes of perinatal depression are preventable [4,5], necessitating the identification of risk factors and behaviors to inform prevention and treatment efforts. Although well-established associations exist between food intake and depression in general populations [6,7,8,9], the limited literature regarding prenatal depression and food intake focuses on macronutrient intake and diet quality [10,11], largely ignoring other important domains of food intake (e.g., direct intake timing, energy intake timing, meal/snack structure, and energy intake distribution across meals).

Chrononutrition refers to the amount, regularity, and timing (ART) of food intake. More specifically, chrononutrition encapsulates one of four health behaviors (i.e., eating, sleep, physical activity, and light/dark exposure) that help entrain the circadian timing system (CST), a biological clock that synchronizes biological processes within the 24 h day cycle [12]. The extant literature identifies several impacts of chrononutrition on facets of cardiovascular health (e.g., obesity, gestational weight gain, type 2 diabetes, and CVD [13,14,15,16,17]), with the emerging literature beginning to investigate the role of chrononutrition indicators in depleted serotonin production, heightened cortisol levels, and disrupted circadian alignment, each leading to consequences including increased risk of depression and anxiety [18].

Moreover, there is current evidence linking specific isolated indicators (e.g., nighttime snacking, eating window, and snack frequency) with postpartum depression [8], consistent with evidence seen in general adult populations [6,7,9]. Further studies have found late-night eating to be associated with delayed melatonin onset and increased systemic inflammation, each contributing to decreased emotional stability [19]. Although this suggests circadian-aligned meal timing as a noninvasive approach that may bolster both metabolic and emotional health [19], a multi-indicator examination of food intake and depression is required to capture the complex nature of dietary patterns and mental health within the perinatal period.

Despite recent interest in chrononutrition and mental health, several critical knowledge gaps remain. (1) The existing literature pertaining to chrononutrition and mental health primarily focuses on child, adolescent, and the general adult populations, largely ignoring perinatal populations [20]. (2) The limited literature that does exist regarding food intake in perinatal populations focuses on the postpartum period and predominately examines dietary quality, nutrient composition (e.g., Mediterranean diet adherence, omega-3 fatty acids, B vitamins, and vitamin D [10,11]), or specific individual timing indicators (i.e., nighttime snacking and breakfast skipping) within postpartum populations [8]. As depression in pregnancy is a strong predictor of worsening or maintained depression during the postpartum period, understanding the role of food intake during the prenatal period is critical [21]. Additionally, with the current understanding that chrononutrition and eating behaviors are more nuanced than macronutrient intake or the timing of isolated eating episodes alone, a broad range of potential food intake indicators that drive prenatal depression, relative to one another, is left unknown.

Thus, this study aims to address research gaps by exploring the relationship between individual mid-pregnancy food intake indicators within five general domains (i.e., intake timing, energy intake distribution across meals, energy intake timing, meal/snack frequency, and overall diet quality) and prenatal depressive symptomatology in order to provide a more comprehensive view of dietary behavior and mental health in pregnancy and generate future hypotheses regarding such associations.

## 2. Materials and Methods

### 2.1. Study Design

This is a secondary data analysis of the Comparison of Two Screening Strategies for Gestational Diabetes (GDM2) trial. This trial was a parallel-arm, single-center, comparative effectiveness trial that compared perinatal outcomes within pregnant individuals that randomly received either a Carpenter–Coustan gestational diabetes (GDM) or the International Associations of Diabetes and Pregnancy Study Groups (IADPSG) screening approach [22,23]. Participants were recruited from 10 different obstetric clinics that were affiliated with the University of Pittsburgh Medical Center Magee-Women’s Hospital from June 2015 to February 2019. The study design and procedure were approved by the University of Pittsburgh Institutional Review Board in accordance with the Declaration of Helsinki. Participants provided written informed consent before enrollment. Oversight was provided by the data and safety monitoring board with the Clinical and Translation Science Institute at the University of Pittsburgh. The GDM2 trial was registered at clinicaltrials.gov (NCT02309138).

Detailed information regarding the protocol for the source dataset can be found in the parent study [22,23]. Participants were recruited between 18 and 28 weeks of gestation, before routine GDM screening. Participants were between 18 and 45 years and had a singleton pregnancy at the time of screening. Exclusion criteria included: (1) diabetes diagnosed <24 weeks of gestation, (2) preexisting type 1 or 2 diabetes mellitus, (3) corticosteroid use in the past 30 days, (4) multiple gestations, (5) hypertension requiring medications, (6) anticipated a preterm delivery due to fetal or maternal indications prior to 34 weeks of gestation, (7) severe liver disease, (8) advanced HIV, or (9) gastric bypass surgery or other conditions that prevented the participant from being administered a glucola solution, (10) major congenital anomaly, and (11) inability to complete glucose testing before 32 weeks gestational age. A total of 921 participants were enrolled within GDM2. This secondary data analysis was limited to participants with data on 24 h food recall and depressive symptoms at base (described in detail below).

### 2.2. Diet Assessment and Food Intake Indicators

Food intake was measured at baseline (visit 1) at 24–28 weeks of gestation using two 24 h dietary recalls on random nonconsecutive weekdays. The Automated Self-Administered 24 h (ASA24^®^) Dietary Assessment Tool was used to collect data; it creates detailed reports on food group consumption and nutrient intake [24]. As the ASA24 asks participants to indicate both what they ate and at what time; energy intake and timing indicators were derived consistently from both recall days. An average of the two assessments was used to calculate food intake indicators within five general domains after each dietary indicator was derived (see Table 1). An eating episode was defined as the consumption of any meal and/or beverage that contained over 50 calories in total, consistent with prior analyses performed on this dataset [15]. Participants reported their own eating episodes and labeled them as breakfast, brunch, lunch, dinner, or a snack; exact label definitions were left for the participant to interpret. We define breakfast, lunch, and dinner as the first, middle, and final main meals of the day, respectively. Brunch is defined as a late-morning meal eaten in addition to or in lieu or breakfast and lunch, while snacks are defined as any energy intake that was not a main meal. One participant with implausible food intake values was excluded from analysis.

### 2.3. Depression

Depression was the main outcome and was measured at baseline (visit 1) at 24–28 weeks of gestation using the Edinburgh Postnatal Depression Scale (EPDS) [25]. The EPDS is a 10-item self-report questionnaire with scores ranging from 0 to 30, with higher scores corresponding with greater depressive symptom severity. Prenatal depressive symptom scores were log-transformed with an offset of 0.5 to manage right-sided skewness. Prenatal depressive symptoms were also dichotomized based on validated clinical cutoffs for moderate (EPDS score of ≥10) and high (EPDS score ≥ 13) depressive symptom severity [25,26,27]. Participants who scored at least a 13 on the EPDS were included in the high depressive symptom group, while participants who scored at least a 10 were included in the moderate to high depressive symptom group. Participants that scored a 13 or above on the EPDS were provided supportive resources for depression. The EPDS has acceptable specificity (78%) and sensitivity (86%) for predicting depression [25] and is well validated for use in pregnant populations [28,29].

### 2.4. Descriptive Characteristics

Demographic characteristics included self-reported age and race (i.e., American Indian/Alaska Native, Asian, Black, Native Hawaiian or Pacific Islander, White, or more than one race). Additionally, participants were asked if they were Latino or Hispanic. Due to small sample sizes, participants were grouped according to the United States census as Black, White, Asian, and “all other racial identities”.

Baseline covariates included psychosocial and behavioral risk factors for prenatal depressive symptoms, as well as factors that could dictate food intake patterns. Participants self-reported employment status (i.e., part-time, full-time, or not working), marital status (i.e., single/divorced, married/living with a partner, or widowed), education (i.e., high school/GED or less, some college/vocational school, college degree, or beyond college), number of adults living within the household, income (i.e., ≤$30,000, $31,000–60,000, or ≥$61,000), current smoking status (i.e., cigarettes, e-cigarettes, or cigars per day), and current alcohol consumption (i.e., yes or no). Self-reported current health status (e.g., poor, fair, good, or excellent) was grouped by poor/fair and good/excellent.

Physical activity was self-reported and measured using the Godin Leisure-Time Exercise Questionnaire, a tool that assesses weekly mild, moderate, and strenuous exercise during leisure time [30]. A Leisure Score Index was computed by summing the frequency of mild, moderate, and strenuous activities and multiplying the type of activity by its respective metabolic equivalent task value.

Insomnia symptom frequency was assessed through self-reporting on how often participants experienced trouble staying asleep, trouble falling asleep, waking several times per night, and waking after their usual amount of sleep feeling tired or worn out in the past month. Scores for each self-report question ranged from 0 (not at all) to 5 (22 to 31 days). Total insomnia symptom frequency was calculated by summing scores across the four items (possible range: 0–20) and was categorized into three equal-width score ranges (0–6, 7–13, and 14–20) because the items were not part of a validated scale with established cut points and the response options represent unequal day-count intervals. Higher scores indicated more frequent insomnia symptoms. One participant with missing insomnia items was excluded from the analysis (complete-case analysis).

Current health status was self-reported as poor, fair, good, or excellent. Participant responses were categorized into two groups: poor/fair and good/excellent. Perceived stress was measured using the Perceived Stress Scale [31], a 14-item scale used to assess the frequency of stress experienced by participants in the past month. Scores range from 0 to 56, with higher scores indicating more perceived stress. Test–retest correlations ranged between 0.55 and 0.85 within the general population [31].

### 2.5. Statistical Analysis

For the primary aim, linear regression was run to examine the association between mid-pregnancy food indicators within five general domains (i.e., direct intake timing, energy intake timing, meal/snack structure, energy intake distribution across meals, and overall diet quality) and total prenatal depressive symptom scores (outcome). Prenatal depressive symptoms were log-transformed with an offset of 0.5 added to account for right skew. All food intake indicators were run within separate models. Relevant covariates that could potentially impact either exposure or outcome variables were added into the main analysis. Covariates were identified based on the conceptual expertise of the research team and included age, income, education, marital status, working status, health status, perceived stress, insomnia frequency, physical activity, and both current alcohol and smoking use. Treatment assignments were also included in the models to preserve the study design of the parent study.

For the secondary aim, Poisson regression was employed within separate adjusted models to examine the association between food intake indicators and moderate to high and high prenatal depressive symptoms. Poisson regression with a sandwich estimator was chosen to produce prevalence ratios (PR) for both ease of interpretation and to avoid overestimation of prevalence that can occur when using logistic regression [32].

Although analyzing multiple indicators increases the risk of Type I error, our study is exploratory in nature; thus corrections were not employed [33]. A post hoc sensitivity analysis was run in the Poisson model for findings that misaligned with hypothesized directions to examine potential impacts of diet quality (specifically the Healthy Eating Index) on initial findings. For all models, exposure variables were mean-centered and standardized to better compare coefficients. Missing data were accounted for using case-wise deletion in both primary and secondary analyses. An alpha level of *p* = 0.05 was deemed statistically significant for hypothesis testing. All analyses were conducted using R (version 4.5.1; R Foundation for Statistical Computing, Vienna, Austria) [34].

## 3. Results

### 3.1. Initial vs. Final Sample Comparison

Of the 921 initial participants enrolled in the GDM2 study, participants with missing (*n* = 204) or implausible (*n* = 1) food intake, covariate, or prenatal depressive symptom variables were excluded from the present analysis, leaving 716 participants (see Supplementary Material). Participants included in the analyses were older, of higher socioeconomic status and educational level, and more likely to be currently married and employed compared with excluded participants. They also had fewer prenatal depressive symptoms and lower perceived stress and were less likely to smoke (see Supplementary Material). Within the included sample, individuals in the high prenatal depressive symptom group reported lower socioeconomic status, lower educational attainment, and lower marriage rates. Additionally, the high prenatal depressive symptom group was more racially and ethnically diverse, had lower employment rates, and had lower self-reported health ratings. Subsequently, the high prenatal depressive symptom group endorsed more insomnia symptoms, slightly lower physical activity levels, and more alcohol/tobacco use. All groups had similar distributions of dietary quality, total energy intake per day, and macronutrient consumption (e.g., carbohydrate, protein, and fat). Detailed sample characteristics by prenatal depressive symptom category (e.g., low, moderate to high, and high) can be found in Table 2.

### 3.2. Main Analysis

**Figure 1:**
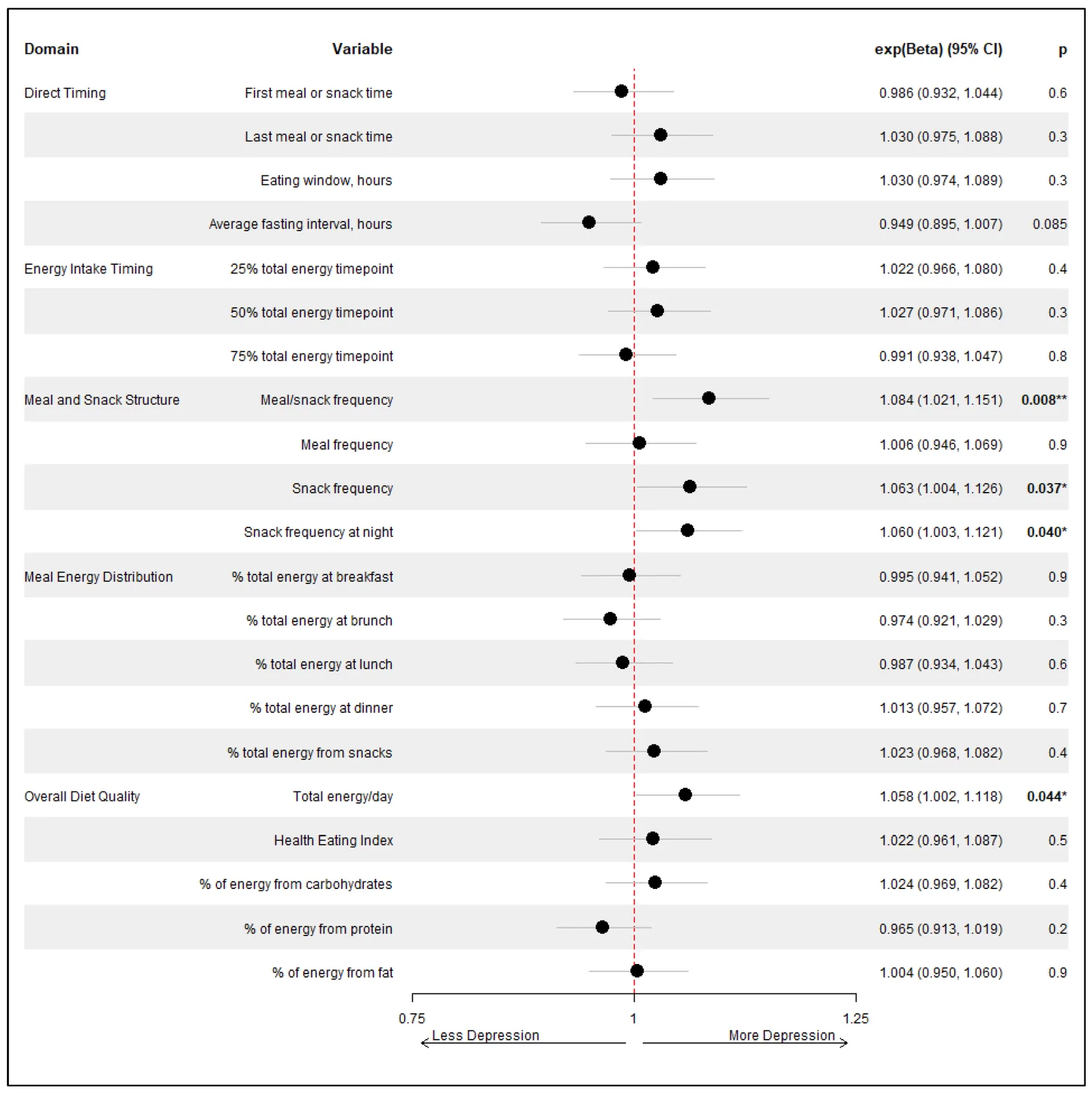
The adjusted associations between food intake indicators and log-transformed prenatal depressive symptoms. Note. 1. * *p* < 0.05, ** *p* < 0.01.

For our main analysis, twenty-one exposure–outcome regressions were performed to explore the relationships between dietary indicators and continuous prenatal depressive symptomatology. In linear regression models, there was a positive relationship between eating frequency and log-depressive symptoms when controlling for covariates (exp(B) = 1.084 [1.021, 0.151], *p* = 0.008), indicating an 8.4% multiplicative increase in average prenatal depressive symptom score for each standard deviation increase in eating episodes. Similarly, there was a positive relationship between log-depressive symptoms and snack frequency (exp(B) = 1.063 [1.004, 1.126], *p* = 0.037), as well as nighttime snacking frequency (exp(B) = 1.060 [1.003, 1.121], *p* = 0.041). There was also a significant positive association for total daily energy intake (exp(B) = 1.058 [1.002, 1.118], *p* = 0.044). Regression results for log-depressive symptoms can be seen in Figure 1.

**Figure 2:**
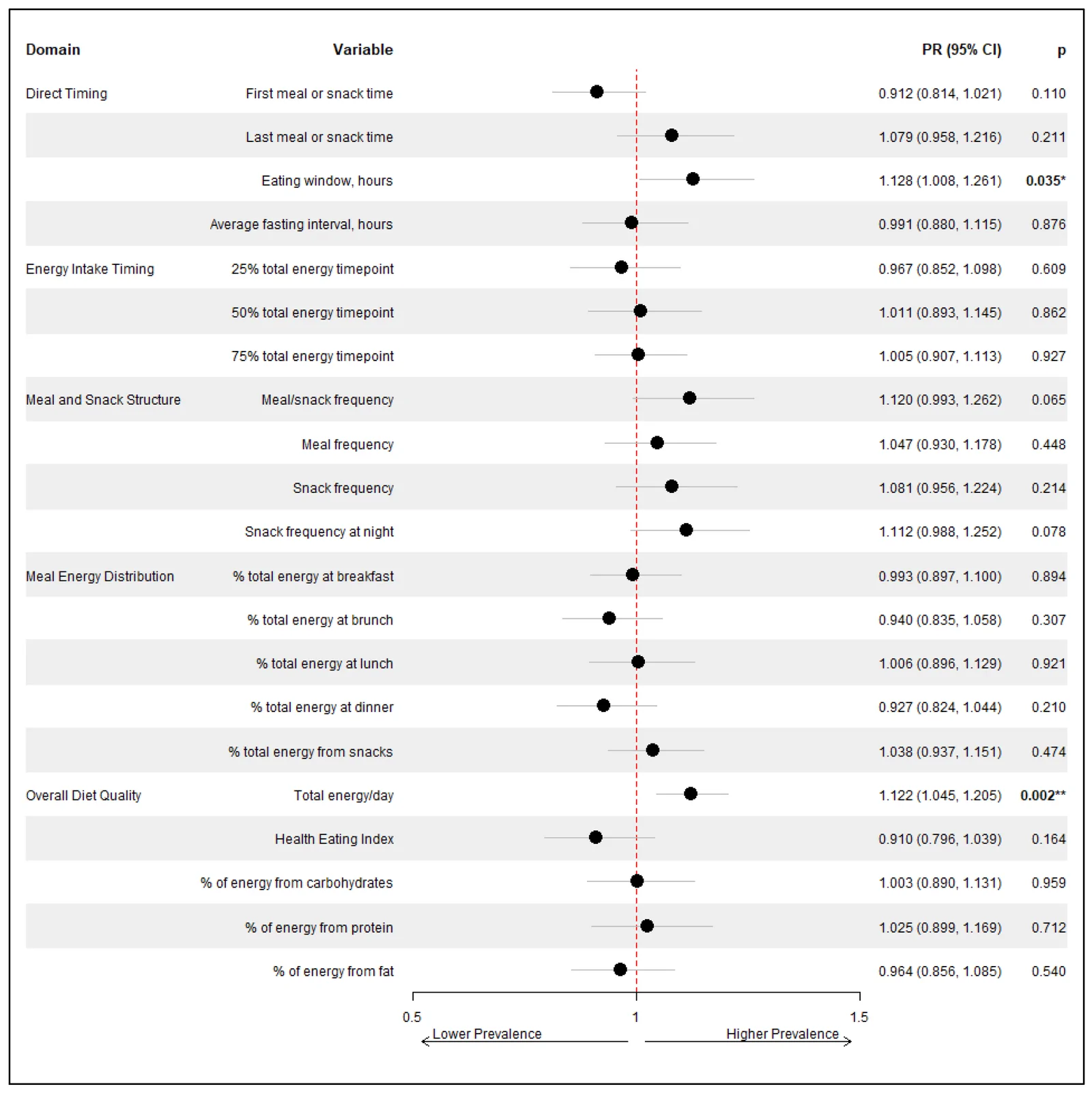
The adjusted associations between food intake indicators and moderate to high prenatal depressive symptoms. Note. 1. * *p* < 0.05, ** *p* < 0.01.

**Figure 3:**
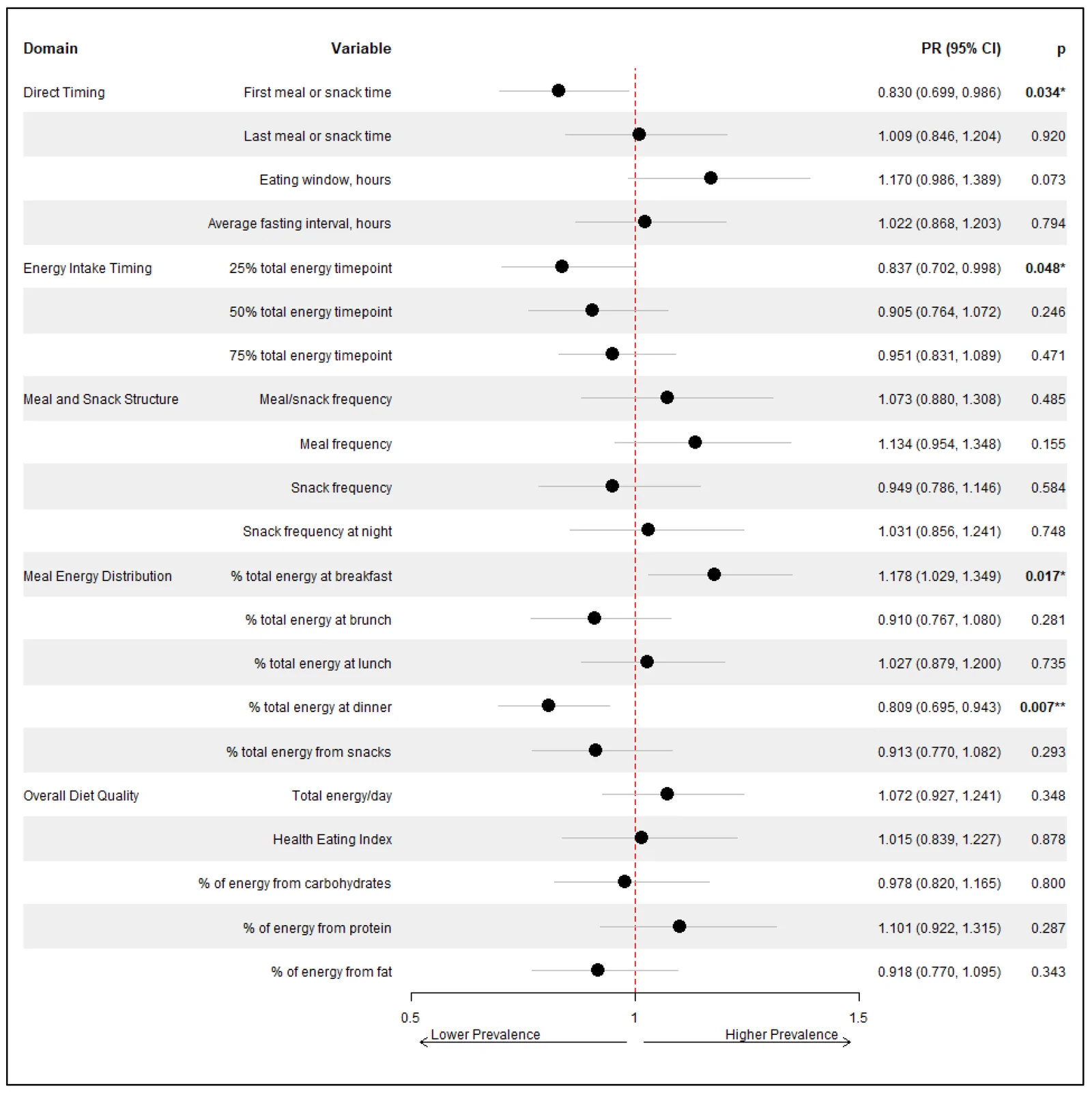
The adjusted associations between food intake indicators and high prenatal depressive symptoms. Note. 1. * *p* < 0.05, ** *p* < 0.01.

For the secondary analysis, twenty-one exposure–outcome regressions were performed to explore the relationships between dietary indicators and moderate to high depressive symptomatology. In the secondary analysis, there was a positive relation between total energy intake and moderate to high prenatal depressive symptoms (PR = 1.122 [1.045, 1.205], *p* = 0.002) after adjusting for covariates, indicating 12.2% higher prevalence in moderate to high prenatal depressive symptoms for every standard deviation increase in energy intake. There was also a significant positive relation between eating window and moderate to high prenatal depressive symptoms (PR = 1.128 [1.008, 1.261], *p* = 0.035). Twenty-one exposure–outcome regressions were again performed to explore the relationships between dietary indicators and high depressive symptomatology. Interestingly, there were several findings that suggested a negative relationship between later food timing and high prenatal depressive symptoms. There was a significant negative relation between the time of the first eating period and high prenatal depressive symptoms (PR = 0.830 [0.699, 0.986], *p* = 0.034), indicating 17% lower prevalence of high prenatal depressive symptoms for each standard deviation increase in first meal or snack time. In addition, there was a negative relation between the time in which 25% total energy was consumed and high prenatal depressive symptoms (PR = 0.837, [0.702, 0.998], *p* = 0.048). There was also a positive relationship between high prenatal depressive symptoms and percentage of energy from breakfast (PR = 1.178 [1.029, 1.349], *p* = 0.017) and a negative relationship between high prenatal depressive symptoms and percentage of total energy at dinner (PR = 0.809 [0.695, 0.943], *p* = 0.007). Post hoc sensitivity analyses in which diet quality was added into the model did not attenuate these results. Robust Poisson regression analyses showing main effects for moderate to high and high prenatal depressive symptoms symptom severity can be seen in Figure 2 and Figure 3, respectively.

## 4. Discussion

This study found positive associations between eating frequency, nighttime snacking frequency, and total prenatal depressive symptoms symptomatology, consistent with previous links between late-night eating episodes and depressive symptoms in both postpartum [8] and general populations. Associations between eating frequency and nighttime snacking frequency may implicate circadian-misaligned eating in prenatal depressive symptoms, which has previously been shown to impact numerous biological mechanisms related to sleep (i.e., nocturnal cortisol response, serotonin and dopamine dysregulation, systemic inflammation, and delayed melatonin production; [19]). Although each of these proposed mechanisms may provide potential rationale for the associations regarding heightened depressive symptomatology within this sample, specific mechanisms related to circadian misalignment (e.g., core body temperature, cortisol wakening response, dim light melatonin onset, etc.) were not examined within this study and should be tested a priori in future research. Additionally, snacking frequency was found to be positively associated with prenatal depressive symptomatology, supporting previous findings in general populations [35]. A 2014 study by Camilleri et al. [36] suggests associations between depression, energy-dense snacking, and emotionally responsive eating in women, a particular facet that was not explored in the present analysis. Within our study, nighttime snacking frequency does not provide insight into calorie density of or macronutrient content in post-dinner food intake. As such, interactions between night-time snacking, emotionally driven eating behavior, and caloric and macronutrient intake in pregnancy warrant further investigation. Due to the cross-sectional nature of this analysis, the possibility that increased prenatal depressive symptomatology may contribute to increased food intake indicators must also be considered.

In addition to meal and snack structure, the current study found positive associations regarding total daily energy consumed and continuous prenatal depressive symptomatology. While the previous literature does not suggest links regarding particular daily caloric thresholds and postpartum depression, excessive gestational weight gain and emotional eating are known to be strong predictors of postpartum depression [37] and should be considered in the future literature when examining total diet quality and prenatal depressive symptomatology.

Further, energy intake, weight, and mood are largely considered bidirectional and cyclical in nature. Although intake of energy-dense foods may offer transient mood improvement, long-term adherence to an energy-dense and high-fat diet has been shown to be associated with weight gain and subsequent negative affective states, creating a self-reinforcing drive to seek further mood improvement through high-calorie food intake [38,39]. Our findings generate further questions, as we did not observe significant findings nor meaningful effect sizes regarding the percentage of energy obtained from fat. Moreover, with the previous literature finding prenatal depression as a predictor for lower maternal diet quality, our null findings regarding several indicators of diet quality and prenatal depressive symptomatology warrant further, longitudinal investigation. It should be noted that the little variability in diet quality within this sample may have decreased the power of our analysis, making it difficult to detect effects. Additionally, the use of a single-point dietary recall assessment may not provide the sensitivity needed in order to comprehensively assess the link between diet quality and prenatal depressive symptoms and is a limitation that should be considered in future studies.

Contrary to the previous literature, results from the main analysis showed positive associations between higher percentage of energy obtained through breakfast and high prenatal depressive symptomatology. These results do not align with previous research showing positive associations between breakfast skipping and depression in general populations [40,41], with the additional literature showing decreased depression risk in prenatal individuals when 5% of total energy obtained from dinner was isocalorically replaced with breakfast-obtained energy [42]. However, our findings do not account for the macronutrient composition of breakfast consumption. Although the recent literature links breakfast quality and depressive symptomatology, implicating the impact of quality breakfast on metabolic and appetite regulation, insulin levels, and blood glucose [43], our results remained unchanged in both effect size and significance after exploring this association with a diet quality indicator introduced into the model during a post hoc sensitivity analysis. Additionally, sleep timing was not considered in the present analysis and should be considered in future research, as it may provide a more robust picture in the context of depression (e.g., depressed individuals may sleep later in the day and report lunch as their first meal, which has impacts on increased energy consumption for participant-defined “breakfast”). These explanations, however, are speculative and should be interpreted with caution. More specifically, future research must test a priori hypotheses involving such proposed mechanisms before suggesting recommendations regarding dietary timing and meal energy distribution.

In addition to the potential implications of macronutrient consumption and sleep timing, our results cannot determine the direction of the relationship between prenatal depressive symptoms and energy obtained through breakfast. Although the previous literature has found emotionally responsive eating to be associated with depressive symptomatology [44,45], it also suggests close associations with night eating syndrome and evening types [46,47,48]. As our findings also suggest that higher energy intake during dinner is associated with lower prevalence of high prenatal depressive symptomatology, these data inadequately explain why positive associations regarding high depressive symptomatology were observed with higher breakfast energy intake and not later meals. Thus, it is critical that future longitudinal studies are conducted to test a priori hypotheses regarding the strength and direction of association regarding prenatal depressive symptoms and the percentage of energy obtained through breakfast.

Additionally, positive associations were found between a later timing of the first eating episode, the first energy intake quartile, and decreased prevalence of high prenatal depressive symptomatology. This does not align with previous research that consistently shows delayed first eating episodes to be associated with poorer physical and mental health outcomes [49]. However, we also found that longer eating windows were associated with moderate to high prenatal depressive symptoms, a result that is supported by the previous literature showing eating windows greater than twelve hours to be positively associated with depression occurrence [40]. Although the previous literature does find associations between delayed or skipped breakfast and depression risk, more current research suggests that meal timing irregularity, and not breakfast skipping, is an independent predictor of postpartum depression [50]. This result, in the context of our significant finding regarding longer eating windows and moderate prenatal depressive symptomatology, may better explain the found associations, although the possibility of Type I error is still present. As our analysis did not consider the consistency or regularity of participant meal times, these results warrant further investigation into the topic when examining food intake timing and prenatal depressive symptomatology. Importantly, these future directions must be tested before any findings can be interpreted to inform dietary timing recommendation.

The significant associations we observed between various domains of food intake, i.e., direct intake timing (e.g., eating window and the timing of the first eating episode), energy intake timing (e.g., the timing of the first energy intake quartile), meal and snack structure (e.g., eating frequency, snack frequency, and nighttime snacking frequency), overall diet quality (e.g., total energy per day), and meal energy distribution (e.g., percentage of energy obtained through breakfast and percentage of energy obtained through dinner), and prenatal depressive symptomatology suggest that food intake is relevant for mood disorders not only in the postpartum period but also during pregnancy. The previous literature suggests that prenatal depression accounts for half of the variance seen in postpartum depressive symptomatology [21]. Considering this, intentional focus should be shifted towards examining, preventing, and treating depression prenatally to buffer severe progression of symptoms after birth, a time that is crucial to a pregnant individual’s physical healing and infant bonding [51,52]. The findings of this study provide insight into modifiable behaviors linking mid-pregnancy dietary patterns with prenatal depression risk and raise questions for future examination. However, it must be noted that although significant, the strength and magnitude of our findings range from small to moderate and may not translate into clinically meaningful effects. Further, all findings are exploratory in nature and should not be interpreted to directly inform dietary nor clinical recommendation, as future research must test a priori hypotheses, establish temporality, and consider potential mechanisms in order to validate findings and implicate any such recommendation.

### Limitations and Future Directions

Although the current study provides a robust foundation to guide future discovery within a diverse and representative sample, limitations must be discussed. First, due to the cross-sectional nature of the analysis, we cannot determine whether (1) food intake indicators caused increased prenatal depressive symptoms, (2) increased prenatal depressive symptomatology caused food intake misalignment, or (3) that this relationship is cyclical, with each factor reinforcing the other. Several factors that might conceptually impact both dietary behavior and prenatal depression (e.g., chronotype, pre-pregnancy BMI, pregnancy-related nausea, and psychiatric history) were not included in this study, and future research may benefit from considering these variables.

Additionally, both primary predictors and outcome measures were obtained through self-reporting, which potentially contributed to shared method variance that might account for portions of the observed associations. Although the EPDS is a well-validated scale and is widely used in studying depression in perinatal populations, using clinical assessments for depression may enhance the robustness of the findings. Similarly, we assessed food intake using a dietary recall within one timepoint in mid-pregnancy, and future research may benefit from assessing dietary intake at various points within pregnancy for a more comprehensive understanding of food intake patterns. Additionally, the use of two dietary recalls on weekdays is not able to capture whether or not the report is representative of a participants’ usual dietary habit and routine [53]. Thus, future research may consider comprehensively measuring multiple reports of dietary intake on weekdays and weekends. Further, capturing participant perspectives on whether their report reflects their normative dietary habits may be relevant for consideration of intake regularity.

It must also be mentioned that only insomnia symptoms were monitored. Insomnia symptom frequency was measured using four items that have not been validated as a composite measure, and the score categories were defined by the possible score range rather than by clinical cut points or sample distribution. Consequently, some participants may have been misclassified, which would likely bias the adjusted estimates toward the null for insomnia and could result in residual confounding by sleep disturbance. A multidimensional assessment of sleep (e.g., timing, regularity, duration, satisfaction, and efficiency) may provide a more robust understanding of how the synergistic nature of circadian-driven behaviors might relate to mental health in pregnancy. Critically, the understanding of dietary intake and prenatal depressive symptomatology would be greatly strengthened by considering the role of sleep timing and multidimensional facets of sleep in future research.

Importantly, although our sample was representative and consisted of individuals with diverse sociodemographic characteristics, it must be acknowledged that participants included within the final analytic sample differed from excluded participants in important ways (e.g., were older, presented higher socioeconomic status, were more likely to be employed and married, and endorsed lower depressive symptoms). This could raise concerns of selection bias, as the analytic sample may underrepresent pregnant individuals with more psychosocial and mental health disadvantage. Additionally, the original sample was recruited through obstetric clinics affiliated with one medical center for comparisons of diabetes screenings, which may impact generalizability of our findings. Future research should consider exploring dietary intake and depressive symptoms with samples retained using more robust recruitment strategies, as prenatal depression and dietary indicators may vary in individuals by region, culture, socioeconomic context, level of access to care, and engagement in health-focused trials.

Lastly, the examination of several indicators increases the likelihood of Type I error, and corrections were not employed due to the exploratory nature of our study [33]. Our findings should be interpreted as a stepping stone to guide future research and generate hypotheses rather than definitive or causal findings. Future research should consider these implications when intending to extend or validate the relationship between food intake and prenatal depressive symptomatology.

## 5. Conclusions

The current study found that eating frequency, snacking, nighttime snacking, and longer eating windows were positively associated with prenatal depressive symptoms within the prenatal period, generating hypotheses regarding circadian misalignment in prenatal psychopathology. Further, our study raises questions regarding the implications of total energy consumed per day, percentage of energy from breakfast and dinner, a later timing of initial energy intake, and the first caloric quartile in prenatal depressive symptomatology. These findings call for future research to explore nuances of macronutrient consumption, energy intake percentages, meal timing regularity, and the possible implications of emotionally responsive eating in the relationship between food intake and prenatal depression.

## Figures and Tables

**Table 1:** Food intake indicators and definitions by domain.

Domain	Indicator	Definition
Direct timing	First eating episode	Time of first intake of energy containing beverage or food
	Last eating episode	Time of last intake of energy containing beverage or food
	Eating window	Time in hours between first and last eating episode
	Fasting interval	Average time in hours between one eating episode and another
Energy intake timing	First energy intake quartile	Time at which 25% of the day’s energy intake was consumed
	Energy intake midpoint	Time at which 50% of the day’s energy intake was consumed
	Third energy intake quartile	Time at which 75% of the day’s energy intake was consumed
Meal and snack structure	Eating frequency	Meal and snack frequency, computed as combined score
	Meal frequency	Number of main meals (i.e., breakfast, brunch, lunch, and dinner)
	Snack frequency	Number of snacks (i.e., calorie consumption not defined as a meal)
	Nighttime snack frequency	Snack frequency occurring after dinner
Meal energy distribution	Breakfast energy intake	Percentage of total energy intake consumed during breakfast
	Brunch energy intake	Percentage of total energy intake consumed during brunch
	Lunch energy intake	Percentage of total energy intake consumed during lunch
	Dinner energy intake	Percentage of total energy intake consumed during dinner
	Snack energy intake	Percentage of total energy intake consumed during snacks
Overall diet quality	Total energy intake	Total energy intake consumed in a day
	Healthy Eating Index	0–100 score derived from 2015 Healthy Eating Index ^1^
	Carbohydrate intake	Percentage of energy obtained from carbohydrates
	Protein intake	Percentage of energy obtained from protein
	Fat intake	Percentage of energy obtained from fat

Note. ^1^ [24].

**Table 2:** Descriptive characteristics by prenatal depression symptom severity.

		Depression Category ^2^		
	Overall	Low DS (<10)	Moderate to High DS (≥10)	High DS (≥13)
Characteristic	*N* = 717 ^1^	*N* = 555 ^1^	*N* = 162 ^1^	*N* = 89 ^1^
Age	29.0 (5.0)	29.4 (4.9)	27.9 (5.2)	27.2 (5.0)
Income level				
≤$30,000	321 (45)	220 (40)	101 (62)	65 (73)
$31,000–60,000	126 (18)	93 (17)	33 (20)	13 (15)
≥$61,000	270 (38)	242 (44)	28 (17)	11 (12)
Education				
High school/GED or less	190 (26)	123 (22)	67 (41)	40 (45)
Some college/vocational school	159 (22)	119 (21)	40 (25)	29 (33)
College degree	191 (27)	154 (28)	37 (23)	14 (16)
Beyond college	177 (25)	159 (29)	18 (11)	6 (6.7)
Marital status	368 (51)	319 (57)	49 (30)	22 (25)
Race				
Black or African American	214 (30)	144 (26)	70 (43)	48 (54)
White	418 (58)	343 (62)	75 (46)	31 (35)
All Other Races	85 (12)	68 (12)	17 (10)	10 (11)
Currently employed	499 (70)	411 (74)	88 (54)	41 (46)
Health status				
Fair/poor	85 (12)	39 (7.0)	46 (28)	29 (33)
Excellent/good	632 (88)	516 (93)	116 (72)	60 (67)
Perceived stress	4.62 (2.77)	3.79 (2.31)	7.48 (2.26)	8.31 (2.27)
Insomnia symptoms frequency				
0–6	343 (48)	290 (52)	53 (33)	21 (24)
7–13	269 (38)	206 (37)	63 (39)	40 (45)
14–20	105 (15)	59 (11)	46 (28)	28 (31)
Godin leisure physical activity score				
<24, active	338 (47)	256 (46)	82 (51)	46 (52)
≥24, moderate or insufficient activity	379 (53)	299 (54)	80 (49)	43 (48)
Current drinkers	22 (3.1)	17 (3.1)	5 (3.1)	4 (4.5)
Current smokers	82 (11)	47 (8.5)	35 (22)	24 (27)
Randomization arm				
Carpenter–Coustan	350 (49)	272 (49)	78 (48)	41 (46)
IADPSG	367 (51)	283 (51)	84 (52)	48 (54)
Depressive symptoms ^2^	6.3 (4.9)	4.19 (2.79)	13.6 (3.3)	15.90 (2.77)
Direct timing				
First meal or snack time	01:28:22 (13:37:57)	08:32:26 (19:43:16)	07:28:53 (19:18:31)	09:10:07 (06:34:43)
Last meal or snack time	17:50:28 (07:16:48)	14:48:39 (10:16:23)	00:13:20 (21:10:01)	22:22:55 (22:06:42)
Eating window, hours	11.69 (2.64)	11.69 (2.51)	11.7 (3.1)	11.4 (3.0)
Average fasting interval, hours	3.39 (1.29)	3.30 (1.17)	3.72 (1.60)	3.91 (1.63)
Energy intake timing				
25% total energy timepoint	18:55:44 (23:18:45)	06:48:39 (15:22:23)	18:13:20 (00:01:02)	11:19:33 (10:28:35)
50% total energy timepoint	21:21:20 (05:32:38)	06:22:42 (00:22:17)	10:00:00 (13:13:00)	14:41:48 (05:31:14)
75% total energy timepoint	05:57:29 (04:20:15)	09:24:19 (23:19:44)	14:35:33 (11:40:48)	01:12:49 (23:36:25)
Meal and snack structure				
Eating frequency	4.86 (1.48)	4.93 (1.46)	4.60 (1.52)	4.31 (1.38)
Meal frequency	3.52 (0.60)	3.57 (0.58)	3.34 (0.66)	3.27 (0.65)
Snack frequency	1.87 (1.11)	1.89 (1.12)	1.77 (1.07)	1.57 (0.95)
Nighttime snacking frequency	0.54 (0.50)	0.53 (0.50)	0.58 (0.52)	0.55 (0.52)
Meal energy distribution				
Breakfast energy intake (%)	21 (12)	21 (11)	21 (14)	24 (15)
Brunch energy intake (%)	3 (11)	3 (11)	4 (12)	4 (12)
Lunch energy intake (%)	28 (13)	28 (13)	28 (16)	28 (16)
Dinner energy intake (%)	34 (14)	34 (13)	35 (16)	35 (15)
Snack energy intake (%)	25 (15)	24 (14)	27 (17)	25 (16)
Overall diet quality				
Total energy intake per day	2132 (1542)	2116 (1598)	2187 (1333)	2026 (1206)
Health Eating Index	54 (9)	55 (9)	51 (8)	51 (8)
Carbohydrate intake (%)	60 (8)	60 (8)	61 (8)	61 (8)
Protein intake (%)	20.2 (5.8)	20.2 (5.7)	20.0 (6.2)	20.1 (6.1)
Fat intake (%)	19.8 (4.4)	19.9 (4.4)	19.3 (4.6)	18.9 (4.5)

Note. ^1^ Mean (SD); *n* (%). ^2^ Depressive symptom categories defined based on Edinburgh Postnatal Depression Scale score. IADPSG = International Association of Diabetes and Pregnancy Study Groups. DS = Depressive symptomatology.

## Data Availability

Data can be made available upon a reasonable request through a data use agreement with the study principal investigator.

## Supplementary Materials

The following supporting information can be downloaded at: https://www.mdpi.com/article/10.3390/nu18213648/s1, Figure S1: Flowchart of sample selection process; Table S1: Characteristics of included/excluded participants.
