# Supplementary material for: Chrononutrition and Prenatal Mental Health: The Relationship Between Food Intake Indicators and Prenatal Depressive Symptomatology
Source: Nutrients. 2026 Nov 2;18(21):3648. doi: 10.3390/nu18213648 (PMC13765481; doi:10.3390/nu18213648)
Supplement: Supplementary file 1 [file nutrients-18-03648-s001.zip › Supplementary_Material/Table_S1.pdf]

Table S1. Characteristics of included/excluded participants

Characteristic	Excluded<br>N = 205[1]	Included<br>N = 716[1]	p-value[23]
Age	27.6 (5.7)	29.0 (5.0)	<0.001***
Income level			<0.001***
≤$30,000	129 (63)	320 (45)	
$31,000-60,000	26 (13)	126 (18)	
≥$61,000	50 (24)	270 (38)	
Education			<0.001***
High school/GED or less	86 (42)	189 (26)	
Some college/vocational school	54 (26)	159 (22)	
College degree	37 (18)	191 (27)	
Beyond college	28 (14)	177 (25)	
Marital status	61 (30)	368 (51)	<0.001***
Race			0.001**
Black or African American	87 (42)	213 (30)	
White	92 (45)	418 (58)	
All Other Races	26 (13)	85 (12)	
Currently employed	122 (60)	499 (70)	0.008**
Health status			0.4
Fair/Poor	17 (15)	85 (12)	
Excellent/Good	96 (85)	631 (88)	
Perceived stress	5.4 (3.2)	4.6 (2.8)	0.016*
Insomnia symptoms frequency			0.035*
0-6	65 (58)	342 (48)	
7-13	28 (25)	269 (38)	
14-20	19 (17)	105 (15)	
Godin leisure physical activity score			>0.9
<24	52 (46)	337 (47)	
≥24	60 (54)	379 (53)	
Current drinkers	4 (3.6)	22 (3.1)	>0.9
Current smokers	32 (29)	82 (11)	<0.001***
Randomization arm			0.2
Carpenter Coustan	111 (54)	349 (49)	
IADPSG	94 (46)	367 (51)	

Characteristic	Excluded<br>N = 205[1]	Included<br>N = 716[1]	p-value[23]
Depressive symptoms	7.9 (6.0)	6.3 (4.9)	0.009**
Moderate Depression (>=10)	35 (31)	162 (23)	0.069
High Depression (>=13)	20 (18)	89 (12)	0.2
First meal or snack time	08:45:04 (07:16:17)	08:35:14 (02:08:43)	>0.9
Last meal or snack time	17:51:00 (04:25:13)	20:17:11 (01:36:07)	0.037*
Eating window, hours	12.35 (5.10)	11.70 (2.63)	0.8
Average fasting interval, hours	6.15 (5.85)	3.39 (1.29)	0.15
25% total energy timepoint	11:47:21 (05:40:28)	11:20:47 (02:15:16)	0.8
50% total energy timepoint	13:15:00 (05:59:04)	14:53:07 (02:24:40)	0.3
75% total energy timepoint	17:13:14 (04:21:06)	18:25:10 (01:55:36)	0.3
Meal/snack frequency	3.21 (2.66)	4.86 (1.48)	0.021*
Meal frequency	2.18 (1.35)	3.52 (0.60)	<0.001***
Snack frequency	1.18 (1.55)	1.87 (1.11)	0.086
Snack frequency at night	0.38 (0.70)	0.54 (0.50)	0.4
% total energy at breakfast	37 (30)	21 (12)	0.13
% total energy at brunch	3 (7)	3 (11)	0.8
% total energy at lunch	51 (40)	28 (13)	0.067
% total energy at dinner	45 (34)	34 (14)	0.3
% total energy from snacks	34 (30)	25 (15)	0.3
Total energy/day	3,767 (7,988)	2,087 (970)	0.4
Health Eating Index	46 (10)	54 (9)	0.002**
% of energy from carbohydrates	51 (11)	60 (8)	0.004**
% of energy from protein	26.1 (10.6)	20.1 (5.7)	0.040*
% of energy from fat	22.9 (3.3)	19.8 (4.4)	0.002**
Age	27.6 (5.7)	29.0 (5.0)	<0.001***

[1]N (%) or mean ± SD

[2]Welch Two Sample t-test; Pearson's Chi-squared test

[3]*p<0.05; **p<0.01; ***p<0.001
